# Why do climate activists stay and why do they leave? Mixed-methods analyses of interview data point to collective efficacy and collective identity beliefs

**DOI:** 10.3389/fpsyg.2026.1863744

**Published:** 2026-10-29

**Authors:** Anna Castiglione, Tal Waltzer, Megan Phelps, Haley Kawar, Adam R. Aron

**Affiliations:** 1 Department of Psychology and Cognitive Science, University of Trento, Trento, Italy; 2 Department of Educational and Counseling Psychology, University at Albany – State University of New York, Albany, NY, United States; 3 Psychology Department, University of California San Diego, La Jolla, CA, United States

**Keywords:** climate action, climate activism, climate change, collective efficacy, collective identity

## Abstract

Climate activist groups play a crucial role in driving policy change, yet they often struggle with small memberships and high attrition. To understand the psychology of what sustains membership in climate organizations, we interviewed activists. We compared those who stayed engaged (*N* = 18) to those who did not (*N* = 17) to find out whether the groups differed in (1) their *reasons for staying engaged* and (2) their *obstacles to staying engaged*. We report two kinds of qualitative analysis (one using bottom-up codes derived from the data and the other using top-down themes from the literature) and one quantitative analysis. We found that, across all three methods, staying engaged was related to various forms of collective efficacy, i.e., the belief that activist groups as a whole can make a difference, the belief that one's particular organization can make a difference, the belief that a particular change making strategy is effective, and the importance of celebrating wins. A second result, also quite consistent across methods, was that those who didn't stay engaged reported a lack of collective identity. This was expressed as weaker alignment with the organization's strategy, weaker community ties, greater interpersonal conflict, and a lack of racial, gender, and class sensitivity and inclusivity. These rich and nuanced analyses of interview data may provide insights to guide organizers to better engage their members.

## 1 Introduction

Global heating is accelerating (Hansen et al., 2025). Without a dramatic reduction in greenhouse gas emissions soon, we risk breaching the critical level of 2 °C between 2040 and 2060 (Masson-Delmotte et al., 2021; Pörtner et al., 2022). In the face of widespread institutional inertia, it is critical for civil society, including grassroots climate activists, to impel policy to cut emissions and to prepare for climate impacts (Fisher, 2024). While grassroots organizations have mounted valiant efforts, their numbers are few and volunteer retention is challenging (Latkin et al., 2023; Gulliver et al., 2023). One way to better understand how to grow and retain membership in these groups is to examine the psychology of collective action.

Social psychology suggests that activist commitment is shaped by a combination of identity-based, moral, and social motivations (Klandermans, 2022; van Stekelenburg and Klandermans, 2023). These include: connection to nature, formative life experiences and a personal sense of mission (Driscoll, 2018), connection to others, shared purpose, and the practices of self- and collective care (Conner et al., 2023). Other research has focused on how activist resilience can be sustained through accommodating individual needs, building a sense of belonging (Drury and Reicher, 2005; Rao and Power, 2021; Gulliver et al., 2023) and showing how a specific movement is effective (Cohen-Eick et al., 2023; Santos Nascimento et al., 2021).

However, while dozens of such studies on collective action have been conducted over the past decade, a minority have specifically examined *climate* activists, whose social struggle has peculiar features (e.g., Buttigieg and Pace, 2013; Fisher, 2016; Fung and Adams, 2017; Wallis and Loy, 2021; Furlong and Vignoles, 2021; Randall and Hoggett, 2019; Gulliver et al., 2023; Ballew et al., 2019; Budziszewska and Głód, 2021). As an example, in-depth interview studies have shown that environmental values, such as non-consumerism, non-wastage, and lifestyle simplicity, as well as self-efficacy, awareness of the climate crisis, experiences in nature, role models, and education are strong motivators for sustained climate engagement (Buttigieg and Pace, 2013). Other qualitative research points to phases of the climate activist trajectory such as “epiphany” (a moment of moral or emotional awakening that motivates engagement), “immersion” (a period of intense involvement and identity integration), “crisis” (experiences of emotional strain, doubt, or burnout), and “resolution” (a reorientation that leads either to sustained engagement or withdrawal) (Randall and Hoggett, 2019). However, because these studies rely primarily on bottom-up approaches by deriving findings solely from interview data, it is difficult to integrate their insights into the broader theoretical literature.

There are however some survey studies that adopt top-down frameworks drawn from existing theory; these have examined associations between climate activism and psychological factors such as social identification (e.g., Furlong and Vignoles, 2021), moral obligation to act (e.g., Wallis and Loy, 2021), and collective efficacy (e.g., Sparks, 2021). But this approach also has a problem—it risks overlooking novel psychological dynamics that may emerge as motivators of collective climate action. Studies employing mixed-methods that try to integrate bottom-up and top-down designs remain rare (see, e.g., Haugestad et al., 2021).

Another weakness in the literature is that most studies of climate activism focus on currently active activists, overlooking those who disengage. A recent exception is Eklöf et al. (2025), who compared active and retired Extinction Rebellion activists and identified barriers such as weak social integration, internal conflict, doubts about impact, burnout, and external stressors, alongside incentives including strong social ties, moral conviction, pragmatic perspectives on impact, and stress-management strategies. However, this study is limited by focusing on a single organization, when climate organizations differ widely.

To return to our original question of understanding how to grow and retain membership in climate activist organizations, we designed a mixed-methods study to examine who stays engaged and who disengages. Specifically, we focus on confirmed current and disengaged climate activists, collect data from them in a structured interview, and discover bottom-up psychological variables from the data while also applying top-down knowledge from the literature. We asked engaged and disengaged activists from several organizations:

(1) What are the most common reasons for staying engaged?
(2) What are the most common obstacles to staying engaged?

We did three kinds of analyses: qualitative bottom-up analysis, quantitative analysis and qualitative top-down analysis, in that order. For the first, we used an inductive, bottom-up coding approach in which codes were refined iteratively in response to patterns emerging from the interview data (Braun and Clarke, 2006; Thomas, 2006), and we report the most commonly mentioned codes which are reasons for staying engaged and obstacles to staying engaged. Although this qualitative bottom-up analysis gives rich data, the large number of codes made it difficult to compare engaged and disengaged groups, and to contextualize the finding within the existing literature. In the second analysis we therefore grouped the codes into a smaller number of psychological factors from an earlier study (Castiglione et al., 2022) and used statistics in a quantitative analysis to test which psychological variables best related to being engaged and disengaged. In a third qualitative top-down analysis, we examined how engaged and disengaged activists differed with regard to four themes that we identified as particularly relevant to climate activism: (1) active hope and the social benefits of activism, (2) practicing organizing for transforming society, (3) the relative benefits of community-based vs. electoral organizing, and (4) individual and group wellbeing. These themes were selected as interpretive lenses based on the authors' long-standing experience with organizing and mobilizing on several justice issues, including climate justice and was also informed by relevant literature on climate activism and social movements. These four themes do not constitute an exhaustive account of the factors relevant to climate activism; rather, they represent a theoretically informed selection of codes that we considered particularly salient in contemporary climate activism.

*Active hope and the social benefits of activism* refers to the idea popularized by Joanna Macy and colleagues (e.g., Macy and Johnstone, 2012) that hope is something we *do* rather than simply *feel* (as opposed to passive, or “optimistic” hope). Active hope is about learning to act regardless of whether one expects to succeed, because acting is the right thing to do, because it teaches us the depths of our hearts, and the ties that bind us (see also Hayes and Kaba, 2023). Our experience that many climate activists explicitly hold these ideas, which sustain them in a struggle against near-impossible odds, is also supported by empirical evidence (Ojala, 2012; Marlon et al., 2019; Dayton et al., 2024).

*Practicing organizing for transforming society* refers to the idea that people engage in climate activism because it is a way to build community-organizing skills that go far beyond just cutting emissions. For example, the skills one learns in this space are essential for addressing a wide range of social struggles, such as energy and food democracy, worker rights, and fighting racism (Marlon et al., 2019; Martiskainen et al., 2020). This linking of struggles has been quite explicit in, for example, the organizing strategy of the youth Sunrise Movement.

*The relative benefit of community-based vs. electoral organizing* refers to the observation that many climate activists favor a theory of change of taking local community action rather than merely expecting the electoral process to be successful. Fisher (2019) showed that young activists combine community and electoral organizing and often discuss the trade-offs of working outside vs. inside “the system,” also see Pickard (2021). Further, organizations such as Sunrise and the Climate Justice Alliance explicitly refer to the limitations of the elite-focused electoral strategy without mass movement pressure.

*Individual and group wellbeing* refers to the awareness that some activists have of moral burnout, overwork and the exhaustion and stress that come from volunteering in spaces where one is constantly exposed to the dread-inducing evidence of climate collapse as well as the obduracy of institutions and political structures. Interview-based evidence supports this awareness among activists (Chen and Gorski, 2015; Randall and Hoggett, 2019), and the training curricula of organizations such as Sunrise and Greenpeace include modules on preventing such activist burnout.

Thus, in what follows we do a qualitative bottom-up analysis to discover the codes that emerge from the interview data, a quantitative analysis to test statistically which factors differ between engaged and disengaged activists and a qualitative top-down analysis to examine the data through the lens of four central themes in climate activism. Our goal is both to integrate these findings with the existing theoretical literature and to identify patterns that can inform activists and organizers seeking to support sustained engagement and participation in climate movements.

## 2 Methods

### 2.1 Participants

Forty activists in California were recruited for this study, which reflects a medium-to-large sample size for qualitative studies (Gubrium et al., 2012). Half of them were engaged activists and the other half were disengaged activists. Among the recruits, five were dropped from the study: three because they did not complete the interview, one because of lack of concentration during the study, and one because of data loss from technical issues with transcription. This resulted in a final sample size of *N* = 35 (*N* = 18 engaged and *N* = 17 disengaged). We focused on young adults (mean age = 24 years, minimum = 18, maximum = 33) because they have historically been strong agents of social and political change. California was selected as the study context because the research team was based there and had established connections with the organizations.

The group was mostly made up of women (68% women, 23% men, 6% non-binary, and 3% other responses) and was ethnically diverse (42% White, 31% Asian, 15% mixed, 9% Hispanic, 3% Black). Most participants identified as Democrats (71%), with the remainder selecting “Other” as their political affiliation (29%, e.g., “Independent,” “Socialist” or “no party affiliation”).

All participants engaged in activism within their organizations as unpaid volunteers.

### 2.2 Recruitment

We collaborated with three major climate activist organizations in California (Sunrise Movement, 350.org, and a University of California climate organization working on environmental advocacy). We distributed a link to a brief eligibility survey through announcements in virtual meetings and events, emails to members in the organizations, and snowball sampling (encouraging those who replied to the survey to pass it on to others).

To be eligible for the study, respondents had to be: 1) either currently active members of a climate organization, defined as having attended at least one meeting per month for the past 3 months (engaged activist), or former active members who had been disengaged for at least 3 months (disengaged activist), 2) proficient in English, and 3) aged 18–35 years old. If inclusion criteria were met, respondents were asked to report their age, gender, income, political orientation, and ethnicity. Eligible participants were then contacted to schedule an interview. Those who completed the interview were paid $30.

### 2.3 Procedure

Each participant had a one-on-one semi-structured interview that was conducted by one of the authors via Zoom, between March and December 2022. Interviews lasted about 1 h (mean = 69.1 min, and SD = 6.16 min) and were audio and video recorded. Participants gave informed consent and all procedures followed a university-approved ethics protocol. The interviewer was trained to make the participant feel comfortable and to encourage them to speak freely. The interview script included questions about the participant's general beliefs about climate change and experiences with activism (see the Supplementary Materials for full script). The main questions in the interview focused on participants' 1) *reasons for staying engaged* and 2) *obstacles to staying engaged* in their organizations. The interview script was identical for engaged and disengaged participants, except for the tense of the questions (e.g., “what keeps you involved with your organization?” for *engaged* participants and “what kept you involved with your organization when you were a member?” for *disengaged* participants).

### 2.4 Data coding for qualitative bottom-up analysis and quantitative analysis

Each interview was automatically transcribed using NVivo and subsequently reviewed and corrected by research assistants for accuracy. Two human coders then segmented the transcripts into distinct statements, each representing an independent paragraph conveying a single concept. The coders categorized each statement as either *meaningful* (i.e., containing information about reasons for or obstacles to staying engaged in activism) or *not meaningful*. In parallel, we developed a preliminary codebook comprising 11 psychological codes underlying pro-environmental behavior, drawing on the environmental psychology literature (Castiglione, 2020; Castiglione et al., 2022) as well as suggestions from experienced California activists whom we surveyed prior to the interviews. The preliminary codebook was intended to guide the initial stages of coding and was subsequently expanded to incorporate new codes emerging from the interview data.

Four human coders were trained on the preliminary codebook and independently coded a subset of the interviews, assigning codes to each meaningful statement. The coders compared their coding and discussed discrepancies to clarify the application and interpretation of existing codes and to identify potential new codes. When coders encountered a potential new code that emerged in at least three interviews, it was added to the codebook through iterative discussion. This process allowed the codebook to be refined through repeated comparison and discussion among coders. All 11 preliminary codes were retained in the final codebook, alongside the additional codes identified through this inductive process. Coding discrepancies were resolved through discussion and consensus among the coders, and the codebook was subsequently “locked.” All 35 interviews were then coded according to the finalized codebook.

The finalized codebook yielded 46 codes for *reasons for staying engaged* (e.g., “concern for the natural world,” “sharing a theory of change with one's organization”) and 20 codes for *obstacles to staying engaged* (e.g., “lack of intersectionality,” “burnout or overwork”). We included only codes mentioned by at least three participants in the final analysis, resulting in a total of 61 codes. This three-participant threshold was used to focus the analysis on recurring patterns rather than idiosyncratic statements in this small interview sample. The full list of 61 codes, together with the frequency with which each code was mentioned, is shown in Table 1. For each interview, we assigned a “1” (present) or “0” (absent) for each code, separately for *reasons for staying engaged* and *obstacles to staying engaged*.

**Table 1:** Bottom-up derived reasons for and obstacles against staying engaged.

Reasons for staying engaged	Description	Number of participants who mentioned code (% total)		
		Total (*N* = 35)	Eng (*N* = 18)	Dis (*N* = 17)
Community	Feeling a sense of belonging or kinship with others in the organization	33 (94%)	17 (94%)	16 (94%)
Moral responsibility	Strong moral commitment to protecting people across the globe, especially the Global South	29 (83%)	14 (78%)	15 (88%)
Vision for the future	Staying motivated because of one's vision for a better future	27 (77%)	14 (78%)	13 (76%)
Sense of meaning	Feeling as though one's climate activism contributes a sense of significance or purpose to one's life	24 (69%)	13 (72%)	11 (65%)
Building transferable skills	Learning practical skills, gaining experience, broadening one's worldview or developing personal strength, organizational and interpersonal skills	23 (66%)	11 (61%)	12 (71%)
Clear roles and ownership	Having defined responsibilities and feeling ownership over specific tasks or areas of responsibility	22 (63%)	11 (61%)	11 (65%)
General collective efficacy	Generally high sense that together, we can make a difference on climate	20 (57%)	10 (56%)	10 (59%)
Reduced climate anxiety	Experiencing less climate anxiety or fear because of involvement in a climate organization	20 (57%)	11 (61%)	9 (53%)
Participative efficacy	Belief that one's actions will contribute to advancing the group's goal	19 (54%)	9 (50%)	10 (59%)
Climate anxiety	Fear or anxiety about climate motivating one's actions	18 (51%)	6 (33%)	12 (71%)
Wanting to do one's part	Taking action for climate because it's the right thing to do, independent of whether it will be successful	18 (51%)	9 (50%)	9 (53%)
Organizational efficacy	Belief in one's organization's ability to successfully make a difference on climate	13 (37%)	6 (33%)	7 (41%)
Homophily	Feeling similar to the group of people one is working with. May include race, class, age, beliefs, gender identity, LGBTQIA+ identification, or political affiliation.	13 (37%)	5 (28%)	8 (47%)
Setting clear boundaries	Scaling back on time commitment of climate activism by setting boundaries, such as not doing activism on specific days or times of the day	13 (37%)	7 (39%)	6 (35%)
Anger and moral outrage	Anger stemming from perceived injustice, triggering engagement	12 (34%)	9 (50%)	3 (18%)
Concern for future of self or close others	Fighting “in one's best interests“, as well as the sense of obligation to care for close others	12 (34%)	4 (22%)	8 (47%)
Fun	Having fun or enjoying one's experience in the organization	12 (34%)	7 (39%)	5 (29%)
Training action competence	Getting trained or mentored by other members of the climate organization to build one's action competence	12 (34%)	10 (56%)	2 (12%)
Intersectionality of org	Staying involved because the organization's projects or mission connect to multiple social issues	11 (31%)	5 (28%)	6 (35%)
Hope	Feeling hopeful regarding the possibility to achieve relevant climate goals with the org's actions	11 (31%)	8 (44%)	3 (18%)
Hopelessness is not constructive	Acknowledgment that hopelessness and doom is not going to help address the climate crisis	11 (31%)	3 (17%)	8 (47%)
Moral concern for global threat	Awareness of global emergency, connected to the obligation to care for the future of humanity	11 (31%)	5 (28%)	6 (35%)
Prognostic frame alignment	Being part of an organization due to agreement with its proposed mitigation strategies	10 (29%)	3 (17%)	7 (41%)
High action competence	A person's ability to successfully take action due to its knowledge of activist know-how	10 (29%)	3 (17%)	7 (41%)
Celebrating wins	Focusing on the organization or movement's successes to increase the sense of collective efficacy	9 (26%)	4 (22%)	5 (29%)
Self care	Practices that one uses to take care of oneself and stay engaged in climate activism	9 (26%)	2 (11%)	7 (41%)
Wanting to make a difference	Motivation to take action to take part in the change making process	9 (26%)	5 (28%)	4 (24%)
Intersectionality of climate	Staying involved because climate intersects with other social issues and identities such as race, gender, or class	8 (23%)	4 (22%)	4 (24%)
General emotional arousal	Being emotionally insipired to be part of the organization/movement	8 (23%)	2 (11%)	6 (35%)
Convenient meetings	Being motivated by meetings that are logistically easy for the participant to attend	8 (23%)	3 (17%)	5 (29%)
Taking breaks	Taking prolonged time off (such as a week or more) to counteract burnout or fatigue	8 (23%)	2 (11%)	6 (35%)
Diverse tasks	Having a variety of tasks to choose from or explore in one's action within the organization	8 (23%)	3 (17%)	5 (29%)
Role in org	Perceiving the importance of one's role in the organization	8 (23%)	4 (22%)	4 (24%)
Concern for the natural world	Feeling worry about climate impacts on the non-human world, feeling responsibility to protect it	7 (20%)	4 (22%)	3 (18%)
Social norm	Believing that other people (whose opinions we value) will support our engagement or will engage as well	7 (20%)	3 (17%)	4 (24%)
Love	Deeply liking one's work inside the org	6 (17%)	3 (17%)	3 (18%)
General self-efficacy	Perceiving that one has competences and skills that can contribute to the success of the organization	6 (17%)	3 (17%)	3 (18%)
Relational organizing	Being tied to an organization thanks to the social relationships embedded in it	5 (14%)	1 (6%)	4 (24%)
Experience of direct climate impacts	Activation because one experiences a climate impact, such as wildfire, flood, drought, etc.	5 (14%)	4 (22%)	1 (6%)
Being a piece of the puzzle	Playing an important role as an individual in the collective action performed by the group	4 (11%)	1 (6%)	3 (18%)
Conflict resolution	Practices employed to reduce or resolve interpersonal conflict	3 (9%)	1 (6%)	2 (12%)
Meeting new people	Enjoying the opportunity to meet new people inside the climate movement	3 (9%)	0 (0%)	3 (18%)
Having enough resources	Feeling like one has enough resources to dedicate to climate activism (e.g., time, and competence)	3 (9%)	3 (17%)	0 (0%)
Place-based organization	An organization that is based in one's local community and thus keeping someone involved	3 (9%)	2 (11%)	1 (6%)
Obstacles to staying engaged	Description	Number of participants that mentioned code (% total)		
		Total (*N* = 35)	Eng (*N* = 18)	Dis (*N* = 17)
Burnout or overwork	Physical and mental fatigue and negative emotions due to over-engagement	23 (66%)	14 (78%)	9 (53%)
Competing responsibilities	Having many responsibilities that compete for time and attention, such as school, work, a family, or a social life	23 (66%)	11 (61%)	12 (71%)
Prognostic frame misalignment	Disagreeing with the strategies that the organization proposes to address climate	12 (34%)	3 (17%)	9 (53%)
Lack of community	Lack of a sense of belonging or kinship with others in the organization	11 (31%)	3 (17%)	8 (47%)
Lack of self-efficacy	The perception of lacking competences and skills that can contribute to the success of the organization	9 (26%)	2 (11%)	7 (41%)
Doomism	Lack of hope, or feeling as though climate breakdown will occur despite our efforts	7 (20%)	1 (6%)	6 (35%)
Climate anxiety	Extreme fear or paralysis due to climate change	6 (17%)	3 (17%)	3 (18%)
Attrition	Mention of other people dropping out of the organization	6 (17%)	5 (28%)	1 (6%)
Interpersonal conflict	Issues arising from disagreements between members of the organization and lack of facilitation inside the group	5 (14%)	0 (0%)	5 (29%)
Loss of collective efficacy	Reduced belief in the ability of the movement to make a difference in addressing climate	5 (14%)	1 (6%)	4 (24%)
Losing	Being unsuccessful in achieving campaign goals	5 (14%)	4 (22%)	1 (6%)
Moving	Moving away from the area in which the climate organization is based	5 (14%)	2 (11%)	3 (18%)
Complexity of organizing	Confusion or overwhelm about the various pathways toward climate action	5 (14%)	3 (17%)	2 (12%)
Emotional engagement	Negative emotions related to working in one's organization	4 (11%)	1 (6%)	3 (18%)
Lack of racial. gender. class sensitivity	Feeling as though the organization does not address racial/gender/class issues competently	4 (11%)	2 (11%)	2 (12%)
Lack of a clear role	Feeling a lack of defined responsibilities or ownership over specific tasks or areas of responsibility	4 (11%)	2 (11%)	2 (12%)
Lack of social norm	Feeling pressured to step away from climate activism by friends, family, or other people	3 (9%)	1 (6%)	2 (12%)
Lack of intersectionality	Leaving because the organization's projects or mission does not connect to multiple social issues	2 (6%)	1 (6%)	1 (6%)

We list all codes arising from the data, ordered by the percentage of all participants mentioning that code (%), and reporting the percentage of engaged and disengaged participants mentioning it. We only report codes mentioned by 3 or more participants.

We did not conduct a formal prospective assessment of thematic saturation. Rather, the coding framework was iteratively refined through coder discussion and comparison until the codebook was locked, with new codes retained when they emerged in at least three interviews (as a criterion used to determine which emergent codes were sufficiently recurrent to be incorporated into the codebook).

### 2.5 Data analysis

#### 2.5.1 Qualitative bottom-up analysis—emerging codes

For both reasons for staying engaged and obstacles to staying engaged, we first calculated the percentage of engaged and disengaged participants who mentioned each of the 61 codes and extracted quotes from within each group for the three most frequent codes.

#### 2.5.2 Quantitative analysis—existing psychological factors

We transposed the 61 codes into nine psychological factors: emotional engagement, collective efficacy, self-efficacy, collective identity, personal identity, perceived behavioral control, social norm, attitude/worldviews, and theory of change, which we had identified in a previous study (Castiglione et al., 2022) (Supplementary Table 1). While some codes are conceptually adjacent, we assigned them according to the factor definitions used in the referenced study, to avoid *post-hoc* double counting. Table 2 shows how we grouped the codes under these nine psychological factors. For each interview we assigned a “1” (present) or “0” (absent) for each psychological factor as follows: if the participant mentioned any of the codes grouped under that factor, we assigned “1,” otherwise, “0.” This binary coding avoided interpreting differences in the frequency of mentions as differences in psychological importance, as these may instead reflect participants' conversational style or the extent to which they elaborated on a topic.

**Table 2:** Mapping of the bottom-up codes to 9 psychological factors.

9 Psychological Factors	Reasons for staying engaged codes	% interviews mentioning code	Obstacles to staying engaged codes	% interviews mentioning code
Attitude/worldviews	Intersectionality of org	31.4	Lack of intersectionality	5.7
	Intersectionality of climate	22.9		
Collective efficacy	General collective efficacy	57.1	Burnout or overwork	65.7
	Organizational efficacy	37.1	Prognostic frame misalignment	34.3
	Prognostic frame alignment	28.6	Attrition	17.1
	Celebrating wins	25.7	Losing	14.3
			Loss of collective efficacy	14.3
Collective identity	Community	94.3	Lack of community	31.4
	Homophily	37.1	Interpersonal conflict	14.3
	Relational organizing	14.3	Lack of racial, gender, class sensitivity	11.4
	Conflict resolution	8.6		
	Meeting new people	8.6		
Emotional engagement	Reduced climate anxiety	57.1	Doomism	20.0
	Climate anxiety	51.4	Climate anxiety	17.1
	Anger and moral outrage	34.3	Complexity of organizing	14.3
	Concern for future of self or close others	34.3	Emotional engagement	11.4
	Fun	34.3		
	Hope	31.4		
	Hopelessness is not constructive	31.4		
	General emotional arousal	22.9		
	Love	17.1		
Perceived behavioral control (PBC)	Convenient meetings	22.9	Competing responsibilities	65.7
	Having enough resources	8.6	Moving	14.3
	Place-based organization	8.6		
Personal identity	Moral responsibility	82.9		
	Sense of meaning	68.6		
	Wanting to do one's part	51.4		
	Setting clear boundaries	37.1		
	Moral concern for global threat	31.4		
	Self care	25.7		
	Taking breaks	22.9		
	Concern for the natural world	20.0		
	Experience of direct climate impacts	14.3		
Self-efficacy	Building transferable skills	65.7	Lack of a clear role	11.4
	Clear roles and ownership	62.9		
	Participative efficacy	54.3		
	Training action competence	34.3		
	High action competence	28.6		
	Wanting to make a difference	25.7		
	Diverse tasks	22.9		
	Role in org	22.9		
	General self-efficacy	17.1		
	Being a piece of the puzzle	11.4		
Social norm	Social norm	20.0	Lack of social norm	8.6
Theory of change	Vision for the future	77.1		

For each of “reasons for staying engaged” and “obstacles to stay engaged,” we list all codes arising from the data, grouped by the 9 psychological factors (italics) and ordered by the percentage of participants mentioning that code (%). We only report codes mentioned by 3 or more participants.

Next, we assessed the reasons for staying engaged on which the engaged and disengaged activists differed. To answer this, we ran nine separate Fisher's exact tests with the dependent variable being whether participants were currently engaged or disengaged, and the independent variable being the percentage of mentions for each psychological factor, and using a Bonferroni-Holm multiple comparisons correction.

For obstacles to staying we took the same approach as above, but this time we ran seven rather than nine Fisher's exact tests, since two of the factors were not mentioned by participants as obstacles: theory of change and personal identity.

The data were analyzed using the R software (R Core Team, 2021).

#### 2.5.3 Qualitative top-down analysis—four central themes in climate activism

We looked at the overall differences between the two groups on the four themes we deemed central to climate activism today, each encompassing multiple emerging themes: 1) Active hope and the social benefits of activism, 2) Practicing organizing for transforming society, 3) The relative benefit of community-based vs. electoral organizing, 4) Individual and group wellbeing.

### 2.6 Reflexivity statement

Several members of the research team, including the authors, have experience organizing within climate and social justice movements. This experience may have shaped recruitment, interview questions, coding, interpretation, and the selection of themes for the top-down analysis. To reduce the influence of individual assumptions, coding involved multiple coders (three of which had no experience in activism), a shared codebook, and discussion of discrepancies. We also acknowledge that the top-down analysis reflects a theoretically informed interpretation shaped by both relevant literature and the researchers' situated experience of activist organizing, rather than an exhaustive or objective account of climate activism.

The interviews were conducted in California between March and December 2022. This timing is relevant to the interpretation of participants' experiences, as the interviews took place in the period following the United States' reentry into the Paris Agreement in 2021, amid continued political contestation over climate policy and active opposition to climate action. We therefore interpret participants' experiences of engagement and disengagement within this particular political and historical context, rather than treating the broader political environment as a uniform or timeless backdrop.

## 3 Results

In this section, we report the findings from the three analyses separately, with the interpretations grounded each analytical approach. Whenever quotes are presented, we use newly generated participant codes rather than the initials stored in the data sheet, to systematically distinguish participants while preserving their privacy and organizational affiliation.

### 3.1 Qualitative bottom-up analysis—emerging codes

#### 3.1.1 The most frequent reasons for staying engaged

The most mentioned reasons for staying engaged, each mentioned by over 70% of participants, were: sense of *community* (94% overall; 94% engaged, 94% disengaged), *moral responsibility* (83% overall; 78% engaged, 88% disengaged), and *vision for the future* (77% overall; 78% engaged, 76% disengaged) (Table 1).

##### 3.1.1.1 Community

The need for community is often expressed as a sense of belonging or kinship with other activists in the organization (Furlong and Vignoles, 2021; Brügger et al., 2020; Fielding et al., 2008; Dono et al., 2010; Bührle and Kimmerle, 2021). Community is also a central element of the well known Social Identity Model of Collective Action (SIMCA; van Zomeren et al., 2008).

For many participants in our study, the social bonds they created with fellow activists were crucial in navigating the emotional and logistical challenges of activism. As one engaged participant explained, the support and solidarity within the activist community helped them cope with the high emotional demands of collective action:

“*I think what really drives me out of it [the hardships of activism] is community, being able to talk with others, being able to work with others in this.”* (eng_01)

Beyond building resilience, community is also a source of positive energy and emotional support. Another engaged participant emphasized the positive effect of being surrounded by people who share their values and vision:

“*It feels good being a part of a community built on the same morals. It's almost like the other way of spiraling—instead of spiraling downward, it's spiraling up.”* (eng_01)

The importance of community emerged also for disengaged participants. Some of them recalled how feeling part of a connected, supportive group had been a key factor in their past involvement. As one participant reflected, without those emotional and social ties, long-term engagement becomes difficult to sustain:

“*It starts with providing a space for people to talk about things, talk about eco anxiety, their own experiences, building a connected community. I just think building community is by far the most important thing in grassroots organizations because we all have kind of the common fight, but there needs to be also something that kind of keeps us together socially, emotionally, and mentally.”* (dis_03)

##### 3.1.1.2 Moral responsibility

Moral responsibility refers to a strong moral commitment to protect people and other living beings affected by the climate crisis. This sense of duty has been identified in prior research as a motivating factor in climate activism, including within movements such as Fridays for Future (Misch et al., 2021; Fisher, 2016; Stuart, 2020; Wallis and Loy, 2021).

Among engaged activists, this moral duty translated into a deep sense of obligation to counterbalance the disproportionate impact of Western countries on all living beings, via land and labor exploitation:

“*I mean, there's so many things that we have and we do that have negative impacts. I think we should all have a sense of personal responsibility. I think that's logical.”* (eng_06)

For some, this sense of responsibility stemmed from their awareness of suffering happening within their own communities, which made inaction simply inconceivable:

“*I would not find it bearable to take an honest look at the world around me, like my immediate community and see all of the pain and suffering and degradation and do nothing about it”* (eng_09).

Others described their moral commitment due to their concern regarding climate impacts:

“*For me, the stakes are too high. I can't just not do anything about it. Not doing anything about it is not an option for me.”* (eng_14)

Disengaged participants also mentioned moral considerations as a central element of their past engagement. For some, the injustice of climate inaction, and particularly its disproportionate effects on future generations, was a key motivator:

“*It's extremely unjust to marginalized communities, to pretend like climate change isn't happening right now just because it's not affecting us. And it's an injustice to our own generation and future generations. And everything we loved and nature and the environment. To not fight for it to be protected when it is very much at risk.”* (dis_07)

##### 3.1.1.3 Having a vision for the future

Many participants were motivated to stay by their vision of a better future. The ability to imagine a positive transformation of the current status quo, such as a more sustainable relationship between humans and the rest of nature, has been shown to support engagement in collective action (Wright et al., 2020, 2022; Clot-Garrell, 2024).

One engaged participant expressed hope toward the growing resistance against fossil fuels:

“*Sure. I mean, I think it's already been started. I'm just looking at what Extinction Rebellion has done, and students on campuses all over. I feel like that fight to really shut down fossil fuel infrastructure has already started.”*

Another activist envisioned a broader transformation:

“*Definitely a carbon tax, definitely valuing ecosystems, especially forests and things that draw down a lot of carbon value. You know, policies that either create tax incentives or force the agricultural sector to invest in carbon drawdown techniques and sustainable agriculture. Subsidizing clean energy and taking away all fossil fuel subsidies.”* (eng_17)

Some found the vision of global systemic change too abstract, and instead focused on tangible, local improvements as the necessary start for greater transformations:

“*I almost want to say that the big thought solutions are more thought exercises and not the small local actions that I really take part in. Not to echo the phrase—think globally, act locally—but that kind of is what it is like. This is the world I want to see on a big scale. So, what do I do in my community to make it better? That way, we can build up to that. What keeps me in activism is the stuff that I can be doing to my local community and the small victories and losses in my local organization.”* (eng_04)

Even disengaged participants mentioned future visions related to community transformation as motivators of their past engagement. One participant described a hopeful vision of social and environmental justice, proposing ideas such as community welfare, shorter work weeks, sharing and repairing to reduce consumption, consistent with degrowth scholarship (Hickel, 2021; Kallis, 2018):

“*Communities where there are more parks so people can like, sit and relax, there's less car centric areas. There is great transportation, not just inside community, but also connecting communities, right? You know, there's a shorter workweek. I don't think we need 5 days. Yeah, I think there's better recycling, there's better education on recycling. What if there was like a service that didn't cost money that could take items from people's houses to other people's houses to make sure we're not putting things in landfill? Maybe if there was more places that you could fix things instead of having to just buy a new one?”* (dis_08)

#### 3.1.2 The most frequent obstacles to staying engaged

The most frequent obstacles to staying engaged were *burnout or overwork* (66% total, 78% engaged, 53% disengaged), *competing responsibilities* (66% total, 61% engaged, 71% disengaged), and *prognostic frame misalignment* (i.e., misalignment with an organization's mission; 34% total, 17% engaged, 53% disengaged) (Table 1).

##### 3.1.2.1 Burnout or overwork

Burnout refers to the physical and mental exhaustion of sustained overwork and/or a sense of moral injury (Arenz, 2023). Within the climate movement, the high stakes of societal and environmental preservation and deep emotional investment can easily turn into overwhelming fatigue, if they are not buffered by a sense of community, solidarity, and belonging (Conner et al., 2023).

Several of our participants pointed out how burnout often arises when volunteer commitment is inconsistent, forcing highly dedicated people in the group to take on a disproportionate share of responsibilities. This unequal burden can create a vicious cycle where overwork leads to burnout, withdrawal, and emotional challenges for those who stay:

“*Volunteers' capacity to help out and volunteer fluctuates. And because of that, it's hard to get consistent commitment. And when you don't have consistent commitment, people have to end up taking on a lot more themselves, who are really, really involved. And so that ends up burning them out.”* (eng_08)

Another frequent source of burnout was the challenge of balancing activism with full-time work in a society that structurally prioritizes professional commitments over civic participation or volunteering. As one participant shared:

“*I'm practically doing this like a part-time job outside of work, so it's difficult to balance life and volunteering and work. When work is already overbearing and stupid in the way that our society is structured, that makes it difficult, like burnout, the burnout makes it difficult.”* (eng_10)

Burnout also appeared in contexts where activism took an *extractive* form, i.e., where the emotional and mental wellbeing of active members was overlooked, and a culture of relentless productivity prevailed:

“*I think it is very easy to fall into traps of burnout. I guess extractive forms of activism itself [plays a role]. Finding oneself in that place of burnout is a very quick pathway to not wanting to be engaged.”* (eng_11)

Multiple disengaged participants shared that the online shift during the COVID-19 pandemic made burnout more likely. During this time, the lack of boundaries in remote work, particularly Zoom meetings, contributed to a sense of overwhelm:

“*I think I was honestly burnt out from doing it, especially because of the whole like, everything's on Zoom. I don't have any boundaries. I could do this literally all day and I would sometimes.”* (dis_08)

Leadership roles were especially linked to burnout, as several disengaged participants described the high pressure of coordinating multiple groups, managing logistics, and maintaining momentum, often with little support:

“*And also, I was a leader the whole time that I was in Sunrise. And I think that's also an extra added layer. From the time I started at 29 until about like a year ago, I was leading two teams. It was just a lot for me.”* (dis_08)

##### 3.1.2.2 Competing responsibilities

The U.S. societal context in which the climate activists we interviewed operate is unforgiving, often demanding full-time work or study while simultaneously managing other responsibilities, such as caring for their family (Castiglione et al., 2022).

Several engaged participants expressed that school and work commitments interfered with their ability to participate in activism. As one mentioned:

“*The time commitment can sometimes be a lot—especially this year, which was my first year back to fully in-person school. I had gotten involved during virtual school, which had a lot more flexibility.”* Another said: “*Personally, I'd say school. Managing such a big responsibility—like getting a degree—while also needing to work to afford to live, that really gets in the way”* (eng_07).

Others described how the pressure of everyone juggling major life responsibilities, and the lack of compensation for activist work (which can be a further challenge to those financially struggling), often led to people leaving, and others to take up their work:

“*Having to balance the things you care about vs. unfortunately, the things that pay the bills. That's tough. We're all giving our time, and our volunteers are giving their time without being compensated, which is something many of us value”* (eng_16).

One disengaged participant explained that their family became a priority when health issues arose, reducing their engagement in climate activism:

“*The biggest reason I quit is because my dad got cancer. That was really stressful, and I just couldn't handle it—worrying about my family and my own health plus the climate stuff at the same time”* (dis_10).

In addition to a lack of time, many reported that feeling exhausted after long days at work made activism feel unsustainable:

“*I'm working full-time and barely have enough energy left to take care of my own basic needs—let alone do mutual aid work or activism”* and “*I got a job—a forty-hour-a-week job—and I just didn't have the time or the energy for it anymore.”* (dis_09)

##### 3.1.2.3 Prognostic frame misalignment

Prognostic frame alignment refers to the way that organizations frame or formulate their strategy for climate mitigation. For example, there is a tension between system-critical framings (“the problem is capitalism”) and those oriented around individual action. According to Wahlström et al. (2013), there are three possible prognostic frames: a) reformist actions of working within the system to develop technological and legislative change; b) individual action that raises awareness but doesn't engage with politics; and c) systemic action which works against the system to end colonialism/capitalism. Disagreements with the solutions an organization proposes can be a significant obstacle to sustained involvement. Some engaged participants noted that different views on whether climate action should address electoral politics, or the degree to which climate justice should be central, can lead to internal tensions:

“*One of the challenges is not seeing eye to eye with people—differences of opinion on politics. Yeah, how much social justice and environmental justice you want to include in our topics.”* (eng_03)

Others expressed frustration with organizations whose leadership feels disconnected from their members, where leadership structures and goals are far from the day-to-day experiences of those doing the work:

“*When big national organizations are not reflective of the people who are putting their blood, sweat, and tears into it, and ignore that—it's just heartbreaking to see.”* (eng_04)

Among disengaged participants, some expressed disillusionment with the limited impact of certain campaigns that focused on narrow issues rather than systemic change:

“*I feel like there are bigger fish to fry. That definitely made it hard for me to stay involved—like, for example, fighting against leaf blowers. That's such a specific issue. I mean, let's focus on corporations, you know what I mean?”* (dis_17)

They became disheartened after engaging in electoral activism, feeling betrayed or let down by the politicians they supported after they gained power:

“*When I first started working in political activism, I genuinely thought I could change the system. It's not that I don't believe in government—it's that politicians suck. For example, I did a lot of work for [local politician]... she claimed to care about our issues, but once she was elected, she didn't care about us anymore. I spent years helping her get elected, and none of the things she said turned out to be true.”* (dis_17)

Engaged and disengaged participants expressed different views on electoral vs. community-based activism, which we discuss in the next section.

### 3.2 Quantitative analysis—existing psychological factors

#### 3.2.1 Reasons for staying engaged

We next examined the differences in the reasons for staying engaged, between engaged and disengaged groups through the lens of the 9 existing psychological factors from a prior study (Castiglione et al., 2022), after grouping our original codes into these factors. Descriptively, the largest difference was observed for *collective efficacy*, which was mentioned by 94% of engaged participants compared with 53% of disengaged participants, a difference of 42 percentage points (Supplementary Figure 1). Although this represented the largest between-group difference, the difference did not remain statistically significant after Bonferroni–Holm correction (*adjusted p* = 0.065; *OR* = 13.9, *95% CI* [1.49–705]). As illustrated in Table 2, our psychological factor of collective efficacy was in fact a construct made up of several codes: the efficacy of collective action in general, the efficacy of the specific organization, the efficacy of the group's particular change-making strategy (“prognostic frame alignment”) and the celebration of wins.

#### 3.2.2 Obstacles to staying engaged

Collective identity showed the largest descriptive difference between groups, with 53% of disengaged participants vs. 22% of engaged participants mentioning this factor, a difference of 31 percentage points (Supplementary Figure 2). However, this difference was not statistically significant (*adjusted p* = 0.601; *OR* = 0.26, *95% CI* [0.04–1.33]). Even so, this trend for collective identity appears informative and we accordingly examine it more carefully. Just as in the collective efficacy case above, the construct of collective identity was made up of several, more detailed codes: i.e., community, interpersonal conflict, and a lack of racial/gender/class sensitivity.

### 3.3 Qualitative top-down analysis—four central themes in climate activism

Here we looked at the overall differences between the two groups on the four themes we deemed central to climate activism today.

#### 3.3.1 Active hope and the social benefits of activism

Recall that active hope refers to acting without necessarily expecting success because of forging the ties that bind, discovering one's courage, being virtuous and being involved in meaningful action. Many engaged activists mentioned that being surrounded by like-minded individuals helped them channel anxiety into action and provided a sense of belonging. One participant said,

“*The community gives me a sense of purpose... it decreases the climate anxiety*.” (eng_08)

Another noted

“*I‘ve met some of my best friends through doing this work, and it's a unique friendship because we're all focused and driven by the same issue*.” (eng_01)

The presence of others doing similar work also reinforced their constructive hope, even if maintaining a realistic sense of the complexity of the struggle: “*I'm not hopeful that our effort will be successful. I'm hopeful that we will act, that we can get together and do as much as we can*” (eng_17). Engaged participants often saw their activism as deeply rooted in personal values and meaning; one said

“*operating off of a personal sense of justice, responsibility and finding meaning… it relates to me the way I see myself, my values and faith*.” (eng_11)

They perceived local and concrete actions directly benefiting their communities as even more meaningful and hopeful compared to actions addressing issues on a larger scale:

‘*I felt hopeless when I was thinking about things on a national level... I have refocused on a more local level, on college campuses—because that's a way that I can kind of overcome the hopelessness issue*.” (eng_06)

Disengaged activists often described a complicated relationship with hope, perhaps indicating they were less familiar with the active hope concept. Many expressed having once felt optimistic and involved but gradually becoming disillusioned by systemic barriers and by the perceived inaction of others. Two people recalled the emotional toll of continuing despite the lack of a sense of progress toward concrete change:

“*I lost hope and was still involved for over a year after that*” and “*I felt so helpless… I was still doing my part, but a part of me was like, I don't care anymore*.” (dis_04)

Although some acknowledged the emotional weight of hopelessness, they tended to manage it through personal rather than collective coping strategies. One participant explained,

“*I don't let [hopelessness] overpower me... I sit with it, I contemplate it, and then I kind of carry on*.” (dis_03)

Another mentioned:

“*When I start to feel it [hopelessness] creeping up, I go sit by the river or tend my garden*.” (dis_09)

Even among those who still hoped that change could be possible, there was a deep skepticism about systemic obstacles, as one person said,

“*I just feel like I'm a very hopeful person….But I'm also like realistic and realize that people's avarice gets in the way and greed of corporations is the biggest obstacle... it's probably not going to happen*.” (dis_16)

Nevertheless, some disengaged activists still found meaning in their past efforts and in climate activism in general:

“*It gave some meaning... maybe you're on the right side of history*” (dis_05)

and in the emotional bond they once felt in the movement

“*we all grieve, we grieve together and we fight together... the struggle is so real*.” (dis_17)

In summary, these results are consistent with our prediction that engaged participants stayed involved through a sense of active hope, finding meaning in their activist community through their shared values, friendships and reciprocal support. On the other hand, disengaged participants felt more isolated and disillusioned, possibly due to their weaker connection to the collective, which led them to rely mostly on personal coping strategies to manage hopelessness.

#### 3.3.2 Practicing organizing for transforming society

Both engaged and disengaged activists acknowledged gaining useful skills that could be carried over to other activist spaces. However, some aspects of how they perceived this differed.

Engaged activists saw their climate organizing as a formative experience, both personally and politically. One participant mentioned how climate spaces shaped their view of leadership and community work:

“*I think it forces me to be better at collaborating, better at problem solving and managing stress”* (eng_02). Another explained, “*Through climate action... I think the kind of learning that I've gotten has been very useful in thinking about just generally how we live in society and civic engagement.”* (eng_11)

Several also described concrete ways in which activism opened up career paths related to climate mitigation, with one saying,

“*That experience also got me [an] internship... working on climate for the mayor's office at the city of Sacramento.”* (eng_11)

The intersectionality of climate and other struggles was central to their discourse, as another said,

“*Once I recognized the intersectionality... it just taught me to learn about the interdisciplinary approaches to different issues.”* (eng_18)

*One* perceived that her group focused on intersectional struggles beyond simple emission reduction:

“*I think climate justice is [what keeps me motivated] because we focus on things other than just straight environmentalism like intersectionality between climate and racial justice, economic justice systems…*”. (eng_02)

Thus, they described learning not just practical skills but also developing a long-term commitment to activism and a cross-sectional understanding of justice.

Disengaged activists presented their experience with climate organizing in more skills-based terms, often describing it as a temporary phase or as something that contributed to other parts of their lives. One gave an overview of the many transferable tools they gained,

“*I think public speaking, writing with a clear message and clear intent. Being able to strategize and have a plan of action. Being able to work with other people and collaborate. Researching, in some words and using data to support.”* (dis_01)

Many mentioned transferring what they learned to their job or other organizations:

“*Just like the process of working with other people and delegating tasks I think has been helpful in the work that I do with other organizations…*”

and

“*It was just kind of a general organizing experience. And that's made me more confident with organizing within my union as well*.” (dis_12)

Others described remaining engaged in climate-related work, in part because of what they had learned in the movement, but via other activist or educational spaces:

“*Still feel like I'm involved, insofar as I think my labor organizing is oriented toward climate activism and so is my teaching. And so everything I'm kind of doing is kind of working toward that goal.”* (dis_02)

Some mentioned being introduced to broader issues like climate justice, feminism, wealth inequality and other intersectional struggles by the climate movement, but others did not feel like these were central in their group predicament, which made them feel alienated:

“*it [the climate organization] basically dodges the climate justice aspect altogether. In many regards, at least I felt that it does*.” (dis_10)

One disengaged activist noted a lack of representativeness of various communities within the climate movement:

“*It's really important to shed light on all the other different necessities of all the different communities and make sure that they're also included in the movement.”* (dis_03)

In summary, as we predicted, engaged participants did perceive their climate organizing as a way to link to other struggles. On the other hand, disengaged activists also gained organizing skills but often described their involvement as temporary, and some felt alienated by a lack of focus on broader justice issues or insufficient inclusivity within their organization.

#### 3.3.3 Electoral vs. community-based organizing

We expected that engaged activists would value community activism more than electoral routes. However, we found that engaged activists tended to express a greater, although sometimes conflicted, commitment to electoral goals as a necessary route to systemic change. They acknowledged the shortcomings of electoral systems, like short-termism, political corruption and limited representativeness; but most still advocated for political engagement to advance climate policy. One engaged activist said:

“*Well, the irony is that I don't trust the current structures of government at all, but I know that we need a democratic government in order to make things work… So there needs to be a concerted political movement to get people who are progressive and want to do what's right into office.”* (eng_04)

Many described change as multifaceted, including political change and community-level change happening in parallel:

“*There's a political layer in which...people vote for politicians that act on climate… and then there's political action that can be taken outside of the scope of our democratic system.”* (eng_05)

and

“*I think you have to tackle this from every different angle... from the individual to the local city level and up to the international level.”* (eng_08)

Others described the concrete work they were doing within political structures, as in:

“*I'm part of the political team... I'm doing a lot of work like with our candidates that we're endorsing who are running... I did a lot of work with the Green New Deal.”* (eng_12)

Many disengaged activists expressed deep skepticism about electoral activism, and considered community-centered, relational, and localized approaches more transformative. They stressed the importance of building resilient local networks, and supporting systems revolving around justice and meeting material needs. One disengaged participant described this as,

“*I am a big fan of prefigurative politics... the world you‘re fighting for can't exist until you start building it in your own life and living by your values with your community.”* (dis_09)

Another stated,

“*I think my approach is like, forget the people at the top. Let's work on just building up a strong community.”* (dis_10)

Another mentioned the importance of “bringing people to a good spot” allowing them to live and afford a healthy life, as a way to indirectly tackle climate change, i.e., transforming the oppressive system that affects everyday people just as much as the climate:

“*working on community level, like increased tree canopies and make sure public transportation was a viable resource to get people to and from places and then like bike lanes…I think I've kind of disregarded the big climate change situation, I think more now about people and how can we get people to be at a good spot*.” (dis_08)

A third rejected electoral approaches completely:

“*I would say that the focus on electoralism... is more or less a waste of time.”* (dis_13)

Others expressed concerns over prioritizing systemic-level climate action before addressing basic community wellbeing, as in:

“*So I think what I would do to mitigate the climate crisis or to get rid of it would be getting more people to realize what's at stake for them and their children and the planet as a whole. But that conversation cannot be had with people who are also struggling to maintain their livelihoods. So by making it more convenient to just exist and to live and pay people like an actual wage, like I just think a lot of things would be better if we cared about people.”* (dis_08)

This suggests that their focus on community and material needs may have shifted their activism away from the climate-specific context, reorienting it toward other more tangible, though still interrelated, issues.

In summary, engaged participants, even if cautious toward the electoral systems, still saw political engagement as an essential leverage to systemic change. Many engaged in multi-level action and were actively involved in both political campaigns or advocacy related to climate policy and community-level organizing. On the other hand, disengaged participants tended to reject electoral activism, and considered it ineffective or corrupt. They focused instead on building resilient, justice-oriented local communities that meet immediate material needs. For them, addressing climate began with supporting people's day-to-day wellbeing, rather than influencing top-down political solutions. Their quotes suggest that some of them might have disengaged from these climate organizations in order to focus on more tangible, social justice issues.

#### 3.3.4 Individual and group wellbeing

Engaged participants mentioned preserving wellbeing by setting personal boundaries, delegating responsibilities, and valuing rest. One engaged activist emphasized the importance of defining personal capacity: “*I'm only one person... so defining my capacity has helped a lot”* (eng_10). Some found empowerment in saying no and focusing their efforts. For example, one engaged participant reflected, “*Not attending meetings, and not feeling sorry for it really helped”* (eng_10), while another shared how her mentor taught her that “*even just say[ing] no to something is self-care for me”* (eng_14). These comments showed a conscious commitment to preserving mental energy and making organizing sustainable. Engaged activists talked about the efforts within their groups to address internal conflict and support constructive communication. One recalled how they raised concerns about a gender gap in leadership and felt listened to and validated:

“*That allowed me to be able to say something and leadership... was like—Yeah, that actually is not a good thing. Let's do something about it.”* (eng_14)

Another noted the importance of “clear boundaries and community agreements” to build a positive environment in her group. Generally, engaged activists framed their organizations as learning environments where both personal growth and community support were possible.

Disengaged activists also expressed setting boundaries as a fundamental strategy to preserve mental health. One person described their strategy as:

“*Personally, I‘ve really been focusing on setting boundaries for myself, like, really understanding how much time I have available, how many responsibilities I can take on. So in order to prevent burnout, I really have to have a raw, honest conversation with myself.”* (dis_03)

For them, speaking to other members about burnout and delegating overwhelming efforts was a key element. One disengaged activist mentioned:

“*When I did experience burnout, it really helped me to just talk about it to the people who I was engaged with”* (dis_03). Another said: “*If I was too busy I'd let them know. And they would help take work off my plate and they were very understanding.”* (dis_10)

When it came to conflict resolution, disengaged activists mentioned more unresolved interpersonal conflict, miscommunication and lack of proper facilitation and representativeness in taking decisions, which led some of them to exit their organization. One participant observed that

“*there's not enough resource and attention on the interpersonal dynamics.”* (dis_04)

Another shared that despite efforts to speak up,

“*When I tried voicing that, I had a concern about it [the decision to support a local politician] I wasn't really given the opportunity to talk about it... there was this urgency of like, well, let's just vote... without really speaking it through.”* (dis_04)

Some described witnessing dynamics where dissent was not welcomed and power was unevenly distributed. One recalled, “*there wasn't that much feeling of an open discussion”* and added that

“*certain people have more power... there was this one person who disagreed... and she got shut down really quick.”* (dis_05)

One further obstacle was seen in the disconnect between the national chapter of the larger organization, and the local chapters:

“*they [the national chapter] would make decisions for hubs without talking to hubs… And there was a lot of miscommunication. There was a lot of disregarding people's [local activists'] very real lived experiences.”* (dis_08)

and the divide between paid and unpaid activists in the organization created further tensions.

In summary, both engaged and disengaged participants recognized the importance of setting personal boundaries and prioritizing wellbeing, but differed in their experience of internal conflict within their activist groups. Engaged participants often mentioned supportive, communicative environments that valued personal capacity, rest, and collective care, and positive experiences of being heard and empowered by inclusive group norms. Disengaged participants encountered more frequent unresolved conflict, top-down decision-making, and a lack of inclusiveness, which made them feel disempowered and unacknowledged, and in some cases led to their departure from activist spaces.

### 3.4 Overview of all key results

Table 3 summarizes the results in order to better guide the following discussion.

**Table 3:** Summary of the results.

Reason/Obstacle	Qualitative bottom-up	Quantitative	Qualitative top-down
Reasons to stay engaged	• Sense of community • Moral responsibility • Vision for future	• *Collective Efficacy (greatest E-D difference)* ° Efficacy of collective action in general ° Efficacy of the specific organization ° Efficacy of the group's particular change-making strategy ° Celebration of wins	• *Active Hope:* the engaged emphasized community and saw it as a core meaning of organizing more than the disengaged • *Organizing to transform society:* the engaged saw their activism as having greater generality to other issues than the disengaged • *Community vs. electoral organizing: t*he engaged appreciated both electoral and community routes, whereas the disengaged were disillusioned with electoral organizing • *Self-care: the engaged described more supportive practices around boundaries and collective care, whereas the disengaged reported more conflict*
Obstacles to staying engaged	• Burnout or overwork • Competing responsibilities • Prognostic frame misalignment	• *Collective Identity (greatest D-E difference)* par ° Community ° Interpersonal conflict ° Lack of racial/gender/class sensitivity	

## 4 Discussion

We interviewed engaged and disengaged climate activists from three organizations in California. We first did a qualitative analysis where we examined the 61 emerging codes, looking at the most frequent reasons for and obstacles to staying engaged in climate activism across all participants. Then we did a quantitative analysis where we mapped the emerging codes onto 9 psychological factors from the prior literature and used quantitative analysis to examine how reasons and obstacles differed between the engaged and disengaged groups. Finally, we compared the two groups on four key themes that we considered central to climate activism today: active hope, practicing organizing for transforming society, electoral vs. community-organizing and individual and group wellbeing.

For the qualitative analysis of codes across all participants, the main reasons to stay involved were community, moral responsibility, and a positive vision for the future, while the main obstacles were burnout or overwork, competing responsibilities, and a lack of alignment with the organization's strategy. For the quantitative analysis, collective efficacy (the belief in the group's power to create change) showed the largest descriptive difference between engaged and disengaged activists among the factors examined, while collective identity showed the largest descriptive difference in relation to obstacles to staying engaged. For the qualitative analysis of the four key themes we observed that engaged activists tended to experience more constructive hope, stronger collective support, and a sense of intersectionality and inclusivity in their groups. They were also more open to combining grassroots work with electoral politics and reported better practices for conflict resolution and collective care. Disengaged activists instead often shifted toward local, community-based organizing related to material needs and sometimes felt alienated after experiencing poor interpersonal dynamics and lack of inclusivity in the climate movement.

The most consistent finding across the analyses was the central role of collective efficacy and a sense of community in sustaining engagement. These themes emerged in the qualitative analysis of bottom-up codes, were reflected in the qualitative analysis of top-down themes, and, in the quantitative analysis, collective efficacy showed the largest observed difference between engaged and disengaged participants. People tend to stay in a given organization when they feel that their group is capable of making a difference and when they have a supportive community that shares their values and their vision for the future. This is in line with accounts of community building as a fundamental ingredient for sustained activism (Tajfel, 1978; Wallis and Loy, 2021; Han, 2014; Ginsburg, 2024). Given that our psychological factor of collective efficacy was in fact a construct made up of several codes, i.e., the efficacy of collective action in general, the efficacy of the specific organization, the efficacy of the group's particular change-making strategy (“prognostic frame alignment”) and the celebration of wins, these results point to different psychological facets of efficacy that organizers can focus on. For example, they could emphasize the importance of their particular change-making strategy, highlight past victories to inspire belief in collective power, celebrate current wins together and build efficient organizational structures with clear roles and even work distribution.

A second notable finding concerned collective identity, which emerged across all three analyses. It appeared in bottom-up codes as a lack of alignment with the organization's strategy, in the quantitative analysis as the largest observed obstacle-related difference between groups, and in the top-down themes through strong social ties and shared meaning among engaged activists, contrasted with alienation linked to poor interpersonal dynamics and lack of inclusivity among disengaged activists. The construct of collective identity was made up of several, more detailed codes: i.e., community, interpersonal conflict, and a lack of racial/gender/class sensitivity. Thus our study provides practical insight to organizers on how to boost these specific elements. For example, collective identity can be supported through group-care activities, conflict-resolution processes, and dedicated inclusion teams that can help bridge differences in class, gender, and age.

Apart from these overarching results, the qualitative analysis of top-down themes also pointed to two other elements that distinguished the engaged from the disengaged: “practicing organizing for transforming society” and views on the value of “community-based vs. electoral organizing.” On the first point, organizers might find ways to emphasize that the skills, thinking and experience of working within their particular organization have greater and wider relevance for transforming society—something that grows in relevance given that we not only have a climate crisis, but a climate, ecological and political crisis, with an authoritarian turn in many places. On the second point, we note that the disengaged expressed less confidence in the electoral route than the engaged and their quotes suggested that some of them might have disengaged from these climate organizations in order to focus on more tangible social justice issues. This is also something that organizers could be aware of—you might be more likely to retain people in your group if you emphasize the local transformational aspects of climate action focussed on people's material interest rather than system-level political change and moral aspirations (Young et al., in review)^1^.

Our findings suggest that disengagement from a particular climate organization should not necessarily be understood as disengagement from activism or collective action more broadly. Several participants classified as disengaged appeared instead to have redirected their energy toward other forms of political and civic engagement, including labor organizing, teaching, mutual aid, or other social justice-oriented local work. Organizational exit may therefore represent movement between forms of collective action rather than withdrawal from collective action itself. This distinction is theoretically important, as it suggests that what appears as disengagement at the organizational level may, in some cases, reflect a shift in the domain, strategy, or social justice focus of participants' activism rather than a decline in their broader commitment to social change.

Many of the above results confirm what was found by Eklöf et al. (2025) in a group of current and retired Extinction Rebellion activists but our study expands those results by examining climate activists across multiple organizations and combining qualitative and quantitative methods, allowing for statistical comparison between engaged and disengaged participants.

### 4.1 Limitations and future considerations

Our study had limitations. First, because it was only focused on activists in three organizations in California in 2022, we cannot know how generalizable our results are. Second, our quantitative analyses should be taken cautiously for two main reasons. A cross-sectional design cannot show that collective efficacy or collective identity are causal to sustained engagement (that would require a longitudinal study with an appropriate experimental manipulation). Also our sample size may have hindered the power to detect group-differences for the psychological factors. Third, our study involved self-selection, in that we interviewed participants who decided to respond to our invitation, rather than selecting at random. Fourth, we had rather too few participants and rather too many codes to use a fully data-driven approach such as factor analysis, or multiple regression. Future studies with larger sample sizes might fruitfully use those approaches to discover other psychological factors than the top-down ones we applied here. Fifth, all participants in our sample were or had been unpaid volunteers within their organizations. Our findings therefore provide limited insight into activists whose organizing constitutes paid employment or who participate in movements with different models of activist labor. This is important because, although volunteer-based activism is usually the largest category of membership in mass organizations in the U.S., it should not be assumed to represent all activism more broadly, particularly across different national contexts (Smith et al., 2022). Future research should examine engagement and disengagement across a broader range of organizational and labor arrangements, including movements in which activism is more closely integrated with paid work, community organizations, or other forms of collective labor. Lastly, our sample was restricted to participants aged 18–35. This age range limits our ability to examine how engagement and disengagement may unfold across longer life-course trajectories or how experiences accumulated over many years shape activists' participation. Future research should include a wider age range to better capture these longer-term dynamics.

## 5 Conclusions

This study pointed to the importance of collective efficacy for those who stay engaged. People may be more likely to stay when they feel that their group is capable of making a difference and when they have a supportive community that shares their values and their vision for the future. Active hope also featured more in those who stay engaged—suggesting they don't only feel that their group is capable of making a difference but that it is worthwhile to struggle even without expecting success but instead for developing one's own courage, skills and finding the ties that bind one to others. Conversely, the study pointed to a lack of collective identity for those who don't stay engaged. This was expressed as a lack of alignment with the organization's strategy, as weaknesses in community, more interpersonal conflict, and a lack of racial/gender/class sensitivity and also a lack of inclusivity in the climate movement. The insights from the rich and nuanced thematic analysis we undertook here, backed up by statistics, may be useful for guiding organizers to better engage their members, and likewise for the wider fields of environmental and climate psychology.

## Data Availability

The datasets presented in this study can be found in online repositories. The names of the repository/repositories and accession number(s) can be found below: Open Science Framework: https://osf.io/7sbw9?view_only=913e8ca3a89f4cdf997ee8cc4713fcf5.

## Footnotes

1 Young, K. A., Dablander, F., and Aron, A. R. (in review). *Growing the Climate Movement by Centering Transformative Adaptation*.
