# Supplementary material for: Why do climate activists stay and why do they leave? Mixed-methods analyses of interview data point to collective efficacy and collective identity beliefs
Source: Front Psychol. 2026 Oct 29;17:1863744. doi: 10.3389/fpsyg.2026.1863744 (PMC13754646; doi:10.3389/fpsyg.2026.1863744)
Supplement: Supplementary file 1 [file Supplementary_file_1.docx]

**Supplementary Materials**

**Interview Scripts**

# *—------------------- Interview Schedule (Group 1: Engaged Participants) -----------------------*

**1. Qualitative interview introduction**

[General Overview of Interview]

**Primary goal:** *The purpose of this interview is to understand the experiences and journeys of climate activists in California. That is, we are interested in understanding who you are and the story of how you got involved in taking action for climate. You don’t have to do anything special; we’ll just be having a casual chat. We’re hoping to be able to see things the way you see them... more like a conversation with a focus on your experience, your opinions and what you think or feel about the topics.*

**2. Verbal consent**

**3. Background Information (5-7 minutes)**

[Overview & Warm-up]

*I would love to begin by first getting to know a bit more about you. For example,*

- Where are you from and when did you move to California?
- What type of work do you do?

**4. General Climate Beliefs (10 minutes)**

*Now, I’m going to ask you some questions about climate change in general.*

- When did you first become aware of climate change?
- What emotions come up when you think about climate change?
- In your opinion, who or what is to blame for climate change?
- In your opinion, what should be done to address climate change?
- What climate organizations have you been part of and in what capacity?

**5a. General Climate Activist Journey (20 minutes)**

*So, the next thing I would like to ask you about today is about how you got involved in climate activism.*

- What has kept you involved in climate activism?
- If you had to think, what are some things that have made staying involved difficult?
- How do you overcome those challenges?
- Does your organization do anything to support you in sustaining your activism?

**5b. Climate Activist Journey in Organization (15 minutes)**

- When did you get involved in your organization? (Was it before COVID or during?)
- Why did you join your particular climate organization?
- What keeps you involved with [org]?
- What are some obstacles to staying engaged with your specific organization?

# *—------------- Activist Interview Schedule (Group 2: Disengaged Participants) ----------------*

**1. Qualitative interview introduction**

[General Overview of Interview]

**Primary goal**: *The purpose of this interview is to understand the experiences and journeys of climate activists in California, including why some people stop taking action for climate. That is, we are interested in understanding who you are and the story of how you got involved in taking action for climate, and also why you stopped taking action. You don’t have to do anything special; we’ll just be having a casual chat. We’re hoping to be able to see things the way you see them... more like a conversation with a focus on your experience, your opinions and what you think or feel about the topics.*

**2. Verbal consent**

**3. Background Information (5-7 minutes)**

[Overview & Warm-up]

*I would love to begin by first getting to know a bit more about you. For example,*

- Where are you from and when did you move to San Diego?
- What type of work do you do?

**4**. **General Climate Beliefs (10 minutes)**

*Now, I’m going to ask you some questions about climate change in general.*

- When did you first become aware of climate change?
- What emotions come up when you think about climate change?
- In your opinion, who or what is to blame for climate change?
- In your opinion, what should be done to address climate change?
- What do you think that individuals should be doing as climate activists to address climate change?
- What climate organizations have you been part of and in what capacity?

**5a. General Climate Activist Journey (20 minutes)**

*So, the next thing I would like to ask you about today is about how you got involved in climate activism.*

- What kept you involved in climate activism when you were active?
- If you had to think, what are some things that made staying involved difficult?
- How did you overcome those challenges?
- Did your organization do anything to support you in sustaining your activism?

**5b. Climate Activist Journey in Organization (15 minutes)**

- When did you get involved in the organization you were part of? (Was it before COVID or during?)
- Why did you join that particular climate organization?
- What kept you involved with [org]?
- What were some obstacles to staying engaged with that specific organization?
- Why are you no longer part of that climate organization?

**Supplementary Table 1:** the 9 psychological factors extracted from the Environmental Psychology literature and their definitions.

| ***9 Psychological Factors*** | ***Psychological factor definition*** |
| --- | --- |
| *Attitude/worldviews* | Whether a person has a positive or negative outlook on climate activism: good/bad, foolish/wise, harmful/beneficial, unpleasant/pleasant, unsatisfying/satisfying, unfavorable/favorable (Fielding et al., 2008), for example based on whether activism is focused on intersectional or individual social justice struggles. |
| *Collective Efficacy* | The belief that one’s group can enact a desired change and that others will join in (Bandura, 2000; Jugert et al., 2016; Roser-Renouf et al., 2014; Lubell 2002). The feeling that one’s group was successful in achieving their goals, and in building collective power. Feeling part of an organization because one believes in the efficacy of its proposed solutions to the climate crisis, i.e. feeling aligned with the group’s prognostic frame: e.g. reformist action, individual action or systemic action (Wahlström et al., 2013). |
| *Collective Identity* | Feeling a tight connection with one’s group or community of activists (Tajfel 1978; Wallis and Loy, 2021). Experiencing a homophilic connection with the group, i.e. feeling similar to others members based on ethnicity, class, age, beliefs, gender identity, LGBTQIA+ identification, or political affiliation. Having tight social relationships within one’s organization (Han, 2020). |
| *Emotional Engagement* | Having a strong affective engagement in relation to the climate crisis, including emotions like fear, worry, risk perception and moral outrage (Barkan, 2004; Roser-Renouf et al., 2014; Weber, 2006). |
| *Perceived Behavioral Control (PBC)* | The control someone feels they have over whether or not they engage in a specific behavior (Ajzen 1991). Feeling personally responsible for whether or not one takes action (e.g. by joining activist events), versus feeling a high instrumental barrier from engaging in action (i.e. lack of time, money, resources to join meetings/actions)(Fielding et al., 2008). |
| *Personal Identity* | Identifying as a “nature protector” and feeling positively about this identity. Having taken other environmental / conservation actions prior to joining a climate organization (Brick et al., 2017). A sense of belonging in nature, referred to one’s experiences in the outdoor world, to understanding their impact on non-human species and the moral responsibility to protect all living beings (Frantz & Mayer, 2004). |
| *Self-Efficacy* | The confidence that one can perform an action and that this action will be successful (Barkan, 2004 & Lubell, 2002). A person’s competence and ease in performing a specific action (i.e. writing a petition, speaking in public, using social media) (Jensen, 1997). The beliefs that one’s actions will critically contribute to advancing the group’s goal (Bamberg et al., 2018). This differs from PBC in that PBC refers to external boundaries that may prevent engagement, while personal efficacy to having the skills/competences to engage. |
| *Social Norm* | What a person believes that other people (whose opinions they value) would think about their pro-environmental behavior (Fielding 2008). Being pushed to (prevented from) taking action, based on the approval (disapproval) from friends/peers/family. |
| *Theory of Change* | How a person believes that action for climate will start and develop: starting from the bottom (e.g. grassroots) and spreading to the top (e.g. government), or starting from the top and trickling down to the bottom (Castiglione, 2020; Mihaylov & Perkins, 2015). This differs from general attitudes and worldviews about the environment and social dynamics, in that Theory of Change reflects a more deliberate, reasoned understanding of how societal transformation is most likely to occur. |

**Supplementary Fig. 1.** Diagram of the percentage of engaged and disengaged activists mentioning each factor as a reason for staying engaged, and table of the percentage of engaged and disengaged activists mentioning each factor as a reason to stay engaged, the factors are ordered by the difference engaged-disengaged.

**REASONS FOR STAYING ENGAGED**

**Engaged versus disengaged**

| **Factor** | **%**  **Engaged (E)** | **%**  **Disengaged (D)** | **%**  **E-D** | ***p*** | **Adjusted *p*** | **OR** | **95% CI** |
| --- | --- | --- | --- | --- | --- | --- | --- |
| **Collective Efficacy** | **94%** | **53%** | **42%** | **.007** | **.065** | **13.97** | **1.49–705** |
| Theory of Change | 83% | 71% | 13% | .443 | 1.00 | 2.04 | 0.32–15.9 |
| Personal Identity | 100% | 88% | 12% | .229 | 1.00 | Inf | 0.20–Inf |
| Collective Identity | 100% | 88% | 12% | .229 | 1.00 | Inf | 0.20–Inf |
| Emotional Engagement | 100% | 88% | 12% | .229 | 1.00 | Inf | 0.20–Inf |
| Self Efficacy | 100% | 88% | 12% | .229 | 1.00 | Inf | 0.20–Inf |
| Attitudes Worldviews | 50% | 41% | 9% | .738 | 1.00 | 1.41 | 0.31–6.67 |
| Perceived Behav Control | 39% | 35% | 4% | 1.00 | 1.00 | 1.16 | 0.24–5.75 |
| Social Norm | 11% | 29% | -18% | .228 | 1.00 | 0.31 | 0.03–2.31 |

**Supplementary Fig. 2. Obstacles to staying engaged.** Diagram of the percentage of engaged and disengaged activists mentioning each factor as an obstacle to stay engaged. Table of the percentage of engaged, and disengaged activists mentioning each factor as an obstacle to stay engaged, the factors are ordered by the difference disengaged-engaged.

**OBSTACLES TO STAYING ENGAGED**

**Disengaged versus Engaged**

| **Factor** | **%**  **Engaged (E)** | **%**  **Disengaged (D)** | **%**  **D-E** | ***p*** | **Adjusted *p*** | **OR** | **95% CI** |
| --- | --- | --- | --- | --- | --- | --- | --- |
| **Collective Identity** | **22%** | **53%** | **31%** | **.086** | **.601** | **0.26** | **0.04–1.33** |
| Self Efficacy | 17% | 41% | 25% | .146 | .878 | 0.30 | 0.04–1.69 |
| Perceived Behavioral Control | 67% | 76% | 10% | .711 | 1.00 | 0.62 | 0.10–3.41 |
| Emotional Engagement | 39% | 47% | 8% | .738 | 1.00 | 0.72 | 0.15–3.34 |
| Social Norm | 6% | 12% | 6% | .603 | 1.00 | 0.45 | 0.01–9.50 |
| Attitudes Worldviews | 6% | 6% | 0% | 1.00 | 1.00 | 0.94 | 0.01–78.4 |
| Collective Efficacy | 83% | 82% | -1% | 1.00 | 1.00 | 1.07 | 0.12–9.38 |
